# The Landscape of Early Growth Response Family Members 1-4 in Hepatocellular Carcinoma: Their Biological Roles and Diagnostic Utility

**DOI:** 10.1155/2022/3144742

**Published:** 2022-08-22

**Authors:** Jinlong Liang, Jingyi Wang, Jinshui Zeng, Zhibo Bai, Zhiyuan Zheng, Yue Zheng, Fengqi Jiang, Di Wu

**Affiliations:** ^1^Department of General Surgery, Xiamen Fifth Hospital, Xiamen, China; ^2^Medical Department, Xiamen Fifth Hospital, Xiamen, China; ^3^Department of General Surgery, Heilongjiang Provincial Hospital, Harbin, China

## Abstract

The incidence of hepatocellular carcinoma (HCC), which is one of the most frequent types of cancer seen all over the world, is steadily growing from year to year. EGR genes are members of the early growth response (EGR) gene family. It has been shown that EGR genes play an increasingly essential role in the development of tumors and the progression of numerous malignancies. However, the possible diagnostic and prognostic roles of EGR genes in HCC have only been examined in a limited number of studies. Expression and methylation data on EGR family members were obtained from TCGA datasets. The prognostic values of EGR members were studied. Additionally, the correlations of EGR members with immune cells were assessed through the single-sample gene set enrichment analysis (ssGSEA). In this study, we found that the expression of EGR1, EGR2, EGR3, and EGR4 was distinctly decreased in HCC specimens compared with nontumor specimens. ROC assays confirmed that they have a strong ability in screening HCC specimens from nontumor specimens. According to the findings of Pearson's correlation, EGR1, EGR2, EGR3, and EGR4 were found to have a negative association with the methylation level. Survival study revealed that EGR1, EGR2, and EGR3 were associated with the clinical outcome of HCC patients. Immune cell enrichment analysis demonstrated that the expressions of all EGR members were positively related to the levels of most types of immune cells, such as macrophages, NK cells, B cells, T cells, eosinophils, and CD8 T cells. Overall, the current work demonstrated the expression mode and prognostic value of EGR members in HCC in a comprehensive manner, offering insights for further research of the EGR family as possible clinical biomarkers in HCC.

## 1. Introduction

Hepatocellular carcinoma (HCC) is a highly predominant malignancy with high mortality, and its incidence has continued to increase worldwide [1]. HCC falls within the category of heterogeneous diseases, and its development is frequently brought on by a number of different etiologies, such as the hepatitis B virus, metabolic syndrome, and long-term usage of alcohol [2, 3]. Despite the fact that many different therapeutic treatments have been applied over the past few years, HCC is still responsible for over 60,000 fatalities and nearly 750,000 diagnoses annually [4, 5]. The poor prognosis of HCC is mostly attributable to the cancer's tendency to spread rapidly inside the liver and to spread to other organs [6, 7]. Few particular biomarkers have been developed and made available for clinical application in diagnosis and prognosis up until this point. Thus, the development of novel biomarkers that have the capability of predicting the outcome of HCC patients is an absolute necessity.

A family of zinc-finger transcription factors called immediate-early gene (IEG) zinc-finger transcription factors is made up of the early growth response (EGR) genes: EGR1, EGR2, EGR3, and EGR4 [8]. IEGs exhibit high amounts of messenger ribonucleic acid (mRNA) transcription within thirty to forty-five minutes of being stimulated, which satisfies the criteria of the term “rapidly activated in response to a stimulus.” [9, 10]. The EGR proteins operate as transcription factors, binding to DNA to control the expression of a large number of genes that are downstream. It is likely that the potential targeting genes play an important role played by EGRs in a variety of biological processes, including memory formation and reconsolidation, synaptic plasticity, inflammation, vascularization, myelination, and growth factor regulation [11, 12]. As a tumor-inhibitor factor, EGR1 exhibited a dysregulated level in several types of tumors [13, 14]. The regulation of the transcription of the heparin enzyme is one of the biological roles that EGR1 performs in tumor cells. Depending on the type of tumor, EGR1 can either play an inhibitory role or an activating role [15]. Lei et al. reported that through the epigenetically mediated silence of DKK1 and the modulation of the Wnt/-catenin pathway, the EGR1-induced overexpression of the lncRNA FOXD2-AS1 contributed to the advancement of hepatocellular carcinoma [16]. In addition to this, researchers have found that EGR2 is only weakly expressed in HCC, and that it can prevent HCC cells from growing, migrating, and invading other cells. This suggests that EGR2 may have an anticancer effect [17]. It was found that the expressions of EGR3 were typically suppressed in HCC specimens and cells. Through the overexpression of Fas ligand, the ectopic expression of EGR3 was able to contribute to the suppression of cell growth and the induction of apoptosis in HCC cells [18]. Similarly, it was also widely reported that EGR4 was implicated in the advancement of a number of different cancers [19, 20]. Based on these findings, EGR members are likely to be crucial regulators in the growth of tumors.

The genetic map of HCC has continued to advance thanks to the discovery of genomics, which has enabled these advancements. Nevertheless, there is a pressing need to find effective gene therapy targets for HCC. Based on recently updated public resources and bioinformatics assays, the expressing profiles and diagnostic values of the EGR family members were exhaustively evaluated in this study.

## 2. Materials and Methods

### 2.1. Data Collection

Both the clinical messages and the gene expression data from the TCGA website (https://portal.gdc.cancer.gov/) were retrieved. The gene expression data were of the form of level 3 RNA-seq FPKM dataset. There were a total of 374 cases of HCC and 50 cases of normal tissue that were downloaded and evaluated.

### 2.2. Comparison of Expressions of the EGR Family in HCC and Nontumor Tissues

The expressions of the EGR family were determined using the HTSeq level 3 data on the genome mRNA expression by the use of the software Perl 5.26. The limma package found in *R* 3.6.0 software was used for the analysis of the differential expressions of members of the EGR family in HCC samples in comparison to nontumor samples. The pheatmap program was applied in order to create a visual representation of the results.

### 2.3. Correlations between mRNA Expressions and Methylation of the EGR Members in HCC

We downloaded data from Illumina Human Methylation 450 K using the GDC Data Transfer Tool, which was approved by TCGA. The data concerned the methylation levels of cg sites in the gene promoter regions of differentially expressed EGR members in HCC tissues. Following that, we made use of the corrplot software to conduct additional research into the relationship between methylation and EGR expressions in HCC. An annotation was performed on the data obtained from Illumina Human Methylation 450 K using the annotation file that can be found on the official Illumina website.

### 2.4. Receiver Operating Characteristic (ROC) Curve Analysis

We determined the diagnostic value of the expressions of EGR members in distinguishing HCC patients by performing a receiver operating curve (ROC) analysis. This allowed us to examine the area under the curve (AUC) value as well as a cutoff value according to the maximum of the Youden index. Finally, we determined the diagnostic value of the expressions of EGR members. The definition of the Youden index is sensitivity plus specificity minus one.

### 2.5. Computational Deconvolution of Infiltrating Immune Cells

We performed the deconvolution analysis using single sample gene set enrichment analysis (ssGSEA) to infer the presence in TCGA-LIHC in order to examine the correlations of the infiltrating immune cell subsets in HCC samples with the expressions of the EGR family. Our goal was to determine whether or not there was a relationship between the two. Spearman's correlation coefficient was used to analyze the associations between the expressions of EGR family members and the abundance scores of immune cells.

### 2.6. Statistical Analysis

All statistical analyses were carried out using *R* 3.6.1 software. *p* < 0.05 was regarded as statistically significant.

## 3. Results

### 3.1. Expression Status of EGR Members and Their Diagnostic Value in HCC Tissues

**Figure 1:**
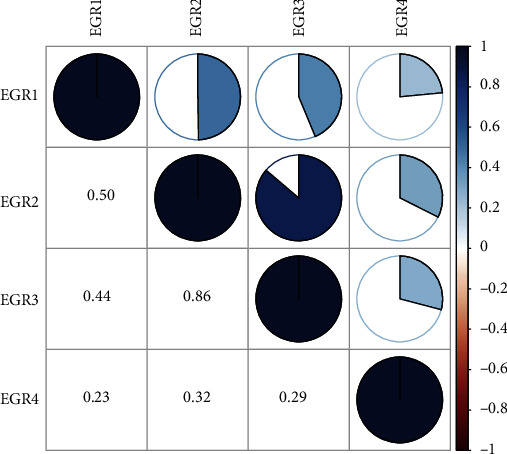
Associations between EGR family members.

Firstly, the mRNA expression data on EGR members (EGR 1-4) from 374 HCC and 50 normal control samples that were received from TCGA were analyzed with Perl software. These samples came from individuals who had been diagnosed with cancer. Pearson's correlation of EGR family genes was determined, and the corrplot software was used to determine whether or not these genes were connected with each other.  Figure 1 demonstrates that there was a meaningful degree of correlation between the genes in the EGR family.

**Figure 2:**
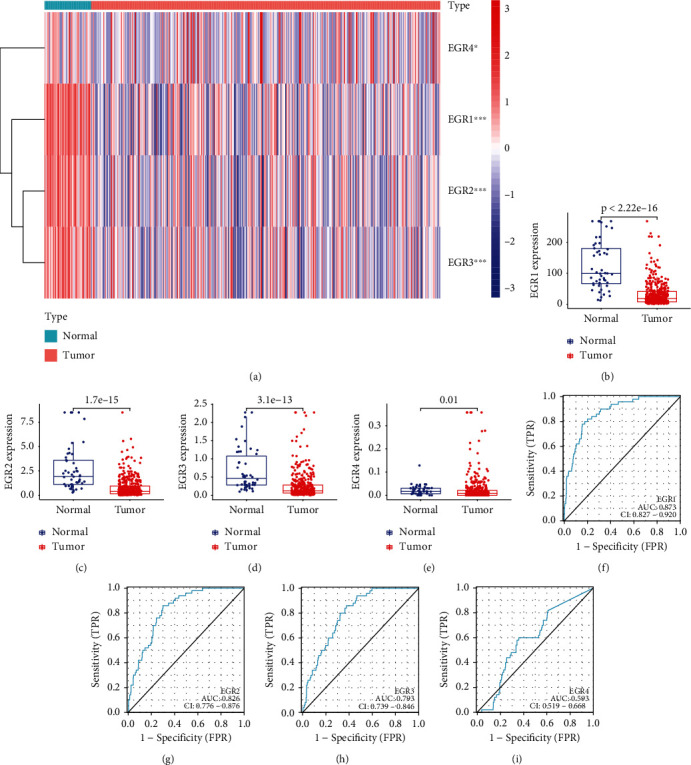
Identification of the dysregulated EGR family members in HCC and their diagnostic value. (a) Heat map showing the expressing pattern of EGR family members between HCC specimens and nontumor specimens. (b)–(e) The expression of EGR1, EGR2, EGR3, and EGR4 was distinctly decreased in HCC specimens compared with nontumor specimens. (f)–(i) The diagnostic value of EGR1, EGR2, EGR3, and EGR4 in screening HCC specimens from normal specimens using ROC analysis.

As exhibited in Figure 2(a), the limma program was applied to evaluate the differentially expressed EGR members, and the pheatmap tool was applied to illustrate the results. We found that the expression of EGR1, EGR2, EGR3, and EGR4 was distinctly decreased in HCC specimens compared with nontumor specimens (Figures 2(b)–2(e)). Then, we explored whether the levels of EGR members had a diagnostic potential. The results of the ROC tests indicated that the low EGR1 expression had an AUC value of 0.873 (95 percent confidence interval: 0.827 to 0.920) for HCC (Figure 2(f)). The low EGR2 expression resulted in an AUC value of 0.826 for HCC, with a 95% confidence interval ranging from 0.776 to 0.876 (Figure 2(g)). The low EGR3 expression resulted in an AUC value of 0.793 for HCC, with a 95% confidence interval ranging from 0.739 to 0.846 (Figure 2(h)). The low EGR4 expression resulted in an AUC value of 0.593 for HCC, with a 95% confidence interval ranging from 0.519 to 0.668 (Figure 2(i)). According to our findings, EGR1, EGR2, and EGR3 could be potential diagnostic criteria for HCC.

### 3.2. Correlation of EGR Expression and Methylation in HCC

**Figure 3:**
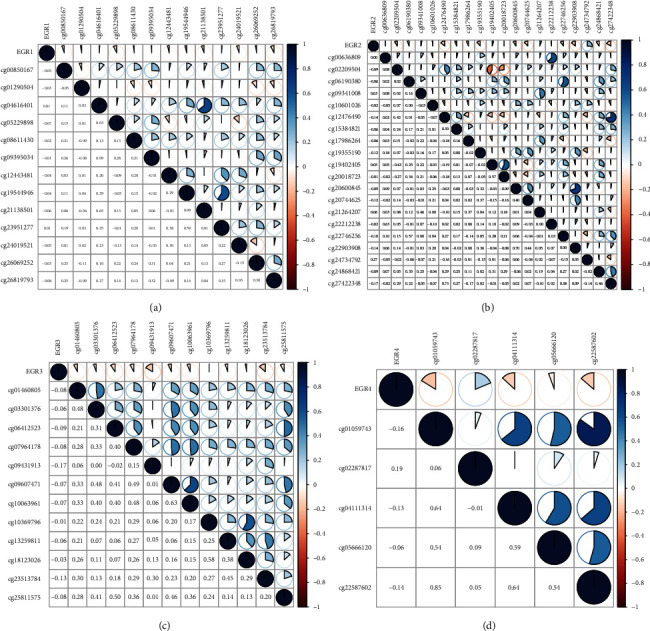
Pearson's correlation between methylation levels and expressions of (a) EGR1, (b) EGR2, (c) EGR3, and (d) EGR4. There was a negative association between the expression level of EGR members and their methylation status in HCC.

One of the most prevalent ways that genes are controlled is through a process known as methylation of their promoter regions. We identified four differentially expressed EGR members in HCC, and they are distinct lowly expressed in HCC specimens. According to the findings of Pearson's correlation, EGR1, EGR2, EGR3, and EGR4 were found to have a negative association with the methylation level (Figures 3(a)–3(d)). According to these findings, there was a negative association between the expression level of EGR members and their methylation status in HCC.

### 3.3. The Prognostic Value of EGR Members in HCC Patients

**Figure 4:**
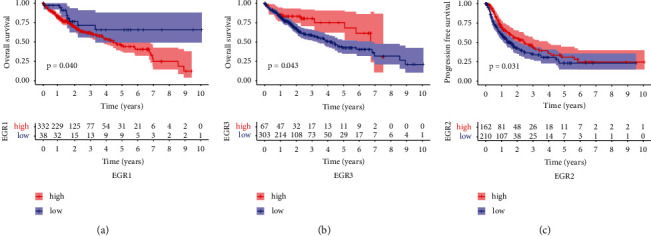
Kaplan-Meier curves estimating the prognostic value of (a) EGR1, (b) EGR3, and (c) EGR2 in patients with HCC.

Kaplan-Meier methods were utilized so that we could investigate the clinical importance of EGR members in HCC patients. As can be seen in Figure 4(a), we found that patients with high EGR1 expression levels displayed lower overall survival rates than patients with low EGR1 expression levels (*p* = 0.040). On the other hand, a high expressions of EGR3 were related to a prolonged overall survival in HCC patients (Figure 4(b)). In addition, our research revealed that individuals with HCC who had low EGR2 expression had a progression-free survival rate that was much lower (*p* = 0.0031, Figure 4(c)). According to the results of our research, EGR1, EGR2, and EGR3 have the potential to serve as a prognostic biomarker for patients with HCC.

### 3.4. Correlation between EGR Members and Tumor Immune Infiltrating Cells

**Figure 5:**
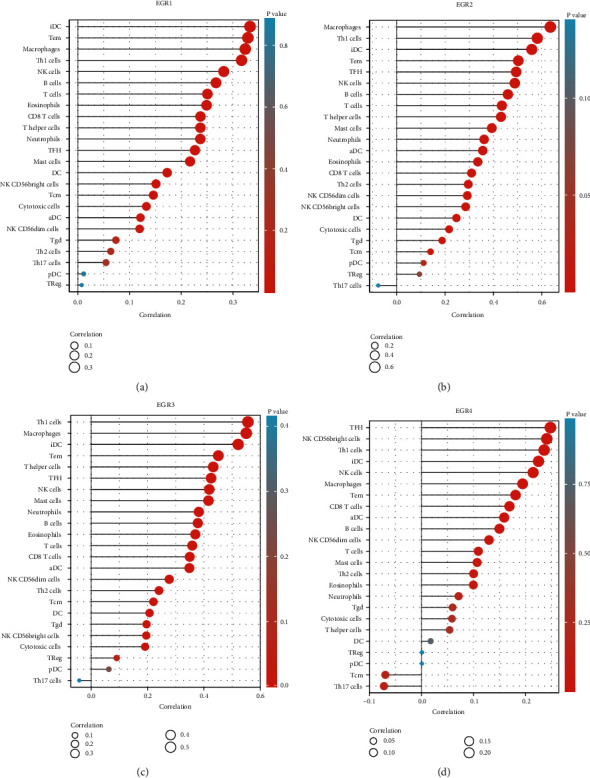
Correlation between EGR family members, including (a) EGR1, (b) EGR2, (c) EGR3, and (d) EGR4, and expression of immune cells (ssGSEA).

We used ssGSEA to investigate the potential immunomodulatory mechanism of EGR members in the regulation of tumor-infiltrating immune cells. Specifically, we were interested in determining whether or not there was a relationship between the expressions of EGR members in TCGA HCC samples and immune infiltrating cells. The data revealed that the expressions of EGR were positively related to the levels of Idc, Tem, macrophages, Th1 cells, NK cells, B cells, T cells, eosinophils, CD8 T cells, T helper cells, neutrophils, TFH, mast cells, DC, NK CD56bright cells, Tcm, cytotoxic cells, aDC, and NK CD56dim cells (Figure 5(a)). The expression of EGR2 was positively correlated with the expression levels of macrophages, Th1 cells, iDC, Tem, TFH, NK cells, B cells, T cells, T helper cells, mast cells, neutrophils, aDC, eosinophils, CD8 T cells, Th2 cells, NK CD56dim cells, NK CD56bright cells, DC, cytotoxic cells, Tgd, Tcm, and pDC (Figure 5(b)). In a manner parallel to this, we discovered that the expression of EGR3 and EGR was favorably related with the majority of different types of immune cells (Figures 5(c) and 5(d)).

## 4. Discussion

Immunotherapy, gene therapy, and molecular targeted therapy are just some of the cutting-edge therapeutic options that are now being researched and developed for HCC [21, 22]. Despite this, the outcomes for patients with HCC remain dismal because there are currently no effective therapeutic approaches [23]. A comprehensive understanding of the molecular processes underlying tumor genesis and progression is necessary for the development of novel prognostic and therapeutic strategies with the goal of improving the prognosis of patients diagnosed with HCC.

Members of the EGR family are garnering an increasing amount of interest as a result of the significant roles they play in cancer [24]. According to accumulating evidence, the EGR family proteins act as tumor suppressor or oncoprotein regulators to control the growth and migration of cells as well as the metabolic process [25, 26]. This and other biological processes like autophagy and their abnormal expression have been confirmed in a variety of cancers. Even though a number of studies have found that members of the EGR family demonstrate dysregulation in HCC, the diagnostic and prognostic relevance of these genes has only rarely been investigated. In this study, to our knowledge, this is the first time that a complete examination of EGR family members based on TCGA datasets has been carried out. We showed that the expression of EGR1, EGR2, EGR3, and EGR4 was significantly lowered in HCC specimens in comparison to nontumor specimens, which suggested that members of the EGR family may serve as a tumor suppressor in the progression of HCC. In addition, ROC assays validated their diagnostic utility by demonstrating that they can distinguish HCC specimens from nontumor tissues. In addition, the EGR1 and EGR3 expression was related with overall survival in HCC patients, whereas the EGR2 expression was connected with progression-free survival in patients with HCC. Our findings were consistent with previous studies.

DNA methylation (DNAm) is one of the epigenetic processes that occurs most frequently [27]. It entails the reversible addition of a methyl group, most frequently to cytosines in the context of CpG dinucleotides, but it does not alter the DNA sequences of the genome [28, 29]. When it comes to cancer diagnosis, the methylation status of DNA is, in general, more trustworthy than the gene expression. In addition, DNA methylation, which serves as the fundamental component of epigenetic changes, plays an important part in the control of cellular processes as well as the development of cancer [30, 31]. Epigenetic modifications in DNA methylation were shown to be relevant to the progression and metastasis of HCC in an increasing number of studies. According to Pearson's correlation, among the four differentially expressed EGR members (EGR1, EGR2, EGR3, and EGR4), the methylation level influences the majority of expression levels, particularly with regard to EGR1 and EGR3. These findings were consistent with earlier researches that demonstrated an inverse relationship between the methylation of EGR and its expression in patients diagnosed with HCC.

Within the immune microenvironment, stromal cells have the ability to alter the carcinogenic properties of tumor cells [32, 33]. Tumor-infiltrating lymphocytes (TILs) are one type of immune cell that plays a significant part in the genesis and progression of cancerous growths [34, 35]. TILs help develop and sustain an immunosuppressive milieu, facilitate immune escape, and ultimately contribute to the progression of tumors by establishing a complex intercellular interaction network [36, 37]. In this study, we discovered that the expression of EGR4, EGR3, EGR2, and EGR1 was positively associated with the expression of a large number of immune cells, which suggested that the enhancement of innate immunity was accompanied by a reduction in adaptive immunity. Additionally, in the tumor microenvironment, invading NK cells and TAMs have high immunosuppressive activities, which decreased the release of IFN- and promotes malfunction in T cells [38]. More and more evidences have confirmed that the existence of gene indicators for CD8 T cells and T helper 1 cells contributes to favorable long-term survivals [39, 40]. Taken together, according to the results of our research, EGR4, EGR3, EGR2, and EGR1 may play an important part in the TME and TICs, and they were strongly associated with immune regulation and the change of the TME.

There are several drawbacks to our study. This study is limited since it is retrospective; hence, there are no data from prospective real-world studies included in it. Second, basic experimental researches were not performed to expand on the results.

## 5. Conclusion

This was the first and most extensive examination of the expressing profiles and clinical importance of EGR members in HCC cases. Our findings could offer a clinically valuable tool for early detection and better prognostic care as well as optimizing the immunotherapy that is related with HCC patients.

## Data Availability

The data used to support the findings of this study are available from the corresponding authors upon request.
